# Acidic Electrolyzed Water Maintains the Storage Quality of Postharvest Wampee Fruit by Activating the Disease Resistance

**DOI:** 10.3390/foods13101556

**Published:** 2024-05-16

**Authors:** Yuzhao Lin, Hongbin Chen, Sisi Dong, Yazhen Chen, Xuanjing Jiang, Yihui Chen

**Affiliations:** 1 College of Oceanology and Food Science, Quanzhou Normal University, Quanzhou 362000, China; yuzhaolin820@163.com (Y.L.); 18392356635@163.com (S.D.); cyz3368@163.com (Y.C.); xuanlikemilk@126.com (X.J.); 2 College of Food Science, Fujian Agriculture and Forestry University, Fuzhou 350002, China

**Keywords:** wampee fruit, storage quality, acidic electrolyzed water, disease resistance, disease resistance metabolism

## Abstract

Harvested wampee fruit is susceptible to disease, resulting in postharvest losses. Acidic electrolyzed water (AEW), a safe and innovative sterilization technology, plays a role in enhancing disease resistance in harvested produce. In this study, the efficacy of AEW in delaying wampee disease development was assessed, along with its association with disease resistance metabolism. Wampee fruit was treated with AEW (pH 2.5) at different available chlorine concentrations (ACCs) (20, 40, 60, and 80 mg/L) and subsequently stored at 25 °C for 8 days. Results revealed that 40 mg/L ACC in AEW (pH 2.5) was most effective in improving the postharvest quality of wampee fruit. Compared with control wampee fruit, those treated with 40 mg/L ACC in AEW exhibited lower incidence of fruit disease, higher pericarp lignin content, and higher activities of pericarp disease resistance enzymes (DREs), such as cinnamate-4-hydroxylase, phenylalanine ammonia-lyase, chitinase, *β*-1,3-glucanase, polyphenol oxidase, 4-coumarate CoA ligase, and cinnamyl alcohol dehydrogenase. These results suggested that AEW elevated DRE activities, promoted lignin accumulation, and ultimately enhanced disease resistance, suppressed disease development, and improved storage quality in harvested wampee fruit. Consequently, AEW emerged as a safe technology to mitigate the disease development and enhance the storage quality of harvested wampee fruit.

## 1. Introduction

Wampee [*Clausena lansium* (Lour.) Skeels], a member of the Rutaceae family, is widely cultivated in southern China, including Guangxi, Guangdong, Hainan, and Fujian Provinces [1,2,3], as well as in other countries such as India, the United States, and Thailand [3,4]. Wampee fruit is rich in nutrients, containing organic acids, polysaccharides, mineral elements, and pectins; therefore, it is well accepted by consumers [1,2]. However, postharvest wampee fruit is susceptible to pathogen invasion, pericarp browning, softening, and quality degradation during storage, negatively impacting the fruit’s quality [1,4]. Notably, postharvest disease occurrence represents a severe decay symptom in wampee fruit. In our observation, the serious disease symptom of wampee fruit began to occur on day 4 after harvest, markedly constraining the quality and shortening the shelf life of the fruit. Consequently, it is imperative to reveal the mechanisms underlying fruit disease development and establish suitable techniques to preserve the quality characteristics of harvested wampee fruit.

Research has indicated that the level of disease resistance can affect postharvest disease development, ultimately compromising the quality of fresh produce [5,6,7,8,9]. Key players in adjusting disease resistance metabolism and influencing disease resistance include disease resistance enzymes (DREs), such as 4-coumarate CoA ligase (4-CL), phenylalanine ammonia-lyase (PAL), *β*-1,3-glucanase (GLU), polyphenol oxidase (PPO), cinnamate-4-hydroxylase (C4H), chitinase (CHI), and cinnamyl alcohol dehydrogenase (CAD), along with disease-resistance-related substances, including lignin [7,10,11,12]. Previous studies showed that the elevated DRE activity and increased disease-resistance-related substance level enhanced disease resistance metabolism, improving disease resistance and suppressing postharvest disease symptoms, thereby preserving fresh product quality [12,13,14,15]. Therefore, maintaining a high level of disease resistance metabolism is crucial for enhancing postharvest disease resistance and improving the quality attributes of fresh products.

Acidic electrolyzed water (AEW) is a safe, novel, convenient, efficient, and cost-effective postharvest handling solution [16,17], characterized by low pH as well as high available chlorine concentration (ACC) and oxidation reduction potential (ORP) [18]. Recognized as the world’s most potent electrolytic water disinfectant, AEW effectively eliminates numerous microorganisms [19,20]. The application of AEW to postharvest fruits and vegetables has been officially permitted in China, the USA, and Japan [16,21]. AEW treatment has been shown to decelerate postharvest disease occurrence, thereby delaying decay development and preserving the quality of fresh produce, such as jujube [20] and longan [8,18]. However, the mechanism underlying AEW-regulated disease resistance and storage quality in harvested wampee fruit via acting on disease resistance metabolism is not fully understood. So, we hypothesized that AEW had a potential ability to inhibit disease development in wampees by regulating disease resistance.

In the present study, we investigated the effects of various AEW concentrations on the quality of harvested wampee fruit and determined the optimal AEW treatment condition for wampee quality. Additionally, we examined the impact of optimal AEW application on the development of fruit disease in harvested wampees. Several critical DREs, including CAD, PAL, C4H, GLU, PPO, 4-CL, and CHI, as well as the levels of disease-resistant substances, such as lignin, were analyzed. To our knowledge, this could be the first report on the mechanism of AEW delaying disease occurrence and maintaining the storage quality of harvested wampees through raising the metabolism of disease resistance. The aims of the present study were to gain insights into the mechanisms underlying AEW-induced disease resistance metabolism, leading to enhanced disease resistance in harvested wampee fruit, and to develop postharvest handling techniques for postponing disease development and improving harvested wampee fruit quality.

## 2. Materials and Methods

### 2.1. AEW Preparation

An AEW machine (HRW-1500, Huo-Ren-Jing-Chuang Medical Equipment Co., Ltd., Beijing, China) was employed to produce AEW. The levels of ORP and ACC were determined through the ORP meter (A57-B, JIA-BEI Water Treatment Co., Ltd., Guangzhou, China) and high-density chlorine meter (RC-3 F, Kasahara Chemical Instruments Corp., Kuki-shi, Japan) severally.

### 2.2. Wampee Fruit and Postharvest Handling

Wampee fruit cv. Jixin was harvested from a wampee orchard (Yongchun County, Fujian, China) within the commercial maturity stage. The postharvest wampees were transported to our laboratory. Then, the wampees with uniformity in color, shape, and size and without diseases or wounds were chosen. These wampees were cleaned with distilled water and used for the next treatment.

A total of 90 wampee fruits were used for estimating the fruit attributes on storage 0 d. The 500 fruits of each group were dipped into the different ACC concentrations of 0 (control), 20, 40, 60, and 80 mg/L of AEW with a pH of 2.5. After 20 min, all treated samples were air-dried for 1 h at 25 °C and packed in a polyethylene film bag of 20 μm thickness (Clorox Enterprise Management Co., Ltd., Guangzhou, China) with a size of 25 cm × 35 cm. Moreover, the osmotic coefficient of moisture, O_2_, and CO_2_ in the bag was about 250 ± 10 g/(m^2^·d·atm), 15.0 ± 0.2 L/(m^2^·d·atm), and 50.0 ± 0.3 L/(m^2^·d·atm), respectively. There were 30 fruits in each bag, stored at 25 °C and 85% relative humidity for 8 d. Throughout storage, 90 fruits from three bags from each group were used every two days to appraise the quality of the wampee fruit, so that the optimal ACC concentration of AEW for enhancing the quality level in wampees might be identified. Additionally, the mechanism by which the AEW treatment at the optimal ACC concentration delayed fruit disease development and maintained the storage quality of wampees, through mediating the disease resistance metabolism, was researched further.

### 2.3. Measurement of Fruit Storability

The rate of commercially acceptable fruit in the wampees was assayed according to the methodology of Lin et al. [22] by counting the percent of 30 fruit samples without diseases and browning on the fruit surface. Furthermore, the methods of prior studies [22,23] were adopted for assaying fruit weight loss percentage. The weight loss percentage was calculated through a comparison with the fruit weight on 0 d. The above results are presented in units of %.

### 2.4. Appraisal of Pericarp Color Variations

As described by Li et al. [18], the values of chromaticity L^*^ and hue angle *h* of the relative four parts of the pericarp equatorial plane in ten wampees were determined using a chromameter (CHROMA METER CR-400, Konica Minolta Inc., Tokyo, Japan).

### 2.5. Evaluation of Pulp Nutrients

The contents of pulp nutrients, such as the titratable acidity (TA), total soluble sugar, total soluble solids (TSSs), and vitamin C of wampees were assayed according to methodologies of prior studies [18,22].

Ten wampees’ pulp was taken to measure the TSS level using a pocket refractometer (PAL-1, Atago Corp., Tokyo, Japan).

Five grams of pulp from ten samples was ground with distilled water; then, the homogenate was filtrated, and the supernatant was acquired. The supernatant (10 mL) was taken to measure the TA amount by an automatic potentiometric titrator (ET18, Mettler Toledo Instrument Co., Ltd., Shanghai, China), with 0.02 mol/L NaOH as the titrant.

One gram of pulp from 10 fruits was ground with 20 mL of distilled water and admixed with 10 mL of 6 mol/L HCl. The mixture was boiled for 30 min; then, one drop phenolphthalein was added. The above mixture was neutralized with 6 mol/L NaOH. The supernatant was acquired for the appraisal of the total soluble sugar level.

One gram of pulp from 10 fruits was ground with 10 mL of 15% (*w/v*) trichloroacetic acid; hereafter, the homogenate was centrifuged, and the supernatant was acquired. One mL of the supernatant was taken to measure the value of vitamin C.

Both vitamin C and total soluble sugar contents are presented in units of g/kg, while both TA and TSS amounts are presented in units of %.

### 2.6. Appraisal of Fruit Disease Index

As described by Li et al. [18], 30 wampee fruits were randomly taken every two days to determine the area proportion of lesions on the fruit surface based on the following five levels: 0, no lesion; 1, 1–24% lesions; 2, 25–49% lesions; 3, 50–74% lesions; 4, ≥75% lesions. The disease index was calculated as Σ (lesion level/the highest level × ratio of corresponding wampee fruit in each level).

### 2.7. Assay of Pericarp Lignin Content

Ten grams of pericarp tissue of wampees were dried at 100 °C to a fixed weight. Thereafter, three grams of dried pericarp tissue were taken for assaying the content of lignin according to the methodology of Sun et al. [7]. Lignin content was expressed as g/kg.

### 2.8. Assay of Pericarp DREs Activities

According to Sun et al. [7] and Tang et al. [8], the activities of DREs (e.g., 4-CL, CHI, PPO, PAL, GLU, CAD, and C4H) were determined.

One gram of pericarp tissue from five wampee fruits was ground with 10 mL of 0.2 mol/L sodium acetate buffer with pH 5.2, including 5 mmol/L *β*-mercaptoethanol, 8% (*w*/*v*) PVP, and 1 mmol/L ethylene diamine tetraacetic acid (EDTA), and then centrifuged. The supernatant was gathered for the appraisal of CHI and GLU activities.

One gram of pericarp tissue from five wampee fruits was ground with 10 mL of 50 mmol/L phosphate-buffered saline (PBS) with pH 5.5, including 2% (*w*/*v*) polyvinyl pyrrolidone (PVP), and then centrifuged. The supernatant was collected for assaying the PPO and PAL levels.

Pericarp tissue (1 g) from 5 wampees was ground with 6 mL of 50 mmol/L Tris-HCl (pH 8.0) including 25% (*v*/*v*) propanetriol and 100 mmol/L dithiothreitol, then centrifuged. The supernatant was gathered to measure 4-CL activity.

One gram pericarp tissue from 5 wampees was ground with 6 mL of 100 mmol/L PBS with pH 6.25 containing 15 mmo/L *β*-mercaptoethanol, 1 mmol/L EDTA, and 0.1% (*w*/*v*) PVP, then centrifuged. The supernatant was collected to measure CAD activity.

One gram pericarp tissue from 5 fruits was ground with 6 mL of 50 mmol/L Tris-HCl with pH 8.9 including 10 μmmol/L leupeptin hemisulfate salt, 0.15% (*w*/*v*) PVP, 15 mmol/L *β*-mercaptoethanol, 4 mmol/L MgCl_2_, 5 mmol/L ascorbic acid, 10% (*v*/*v*) propanetriol, and 1 mmol/L phenylmethylsulfonyl fluoride, then centrifuged. The supernatant was collected to assay C4H activity.

Additionally, the protein levels of the above enzyme solutions were determined as described by Bradford [24]. DRE activities were expressed as U/kg.

### 2.9. Statistic Analysis

In the present work, the parameters were determined three times. Data in the figures are presented as the mean ± standard error (*n* = 3). The SPSS software (version 21.0, IBM Corp., New York, NY, USA) was adopted for analyzing the experiment data based on the analysis of variance as well as Duncan’s tests. The difference at *p* < 0.05 or *p* < 0.01 was treated as the significance level.

## 3. Results

### 3.1. Changes in Fruit Appearance Quality

**Figure 1:**
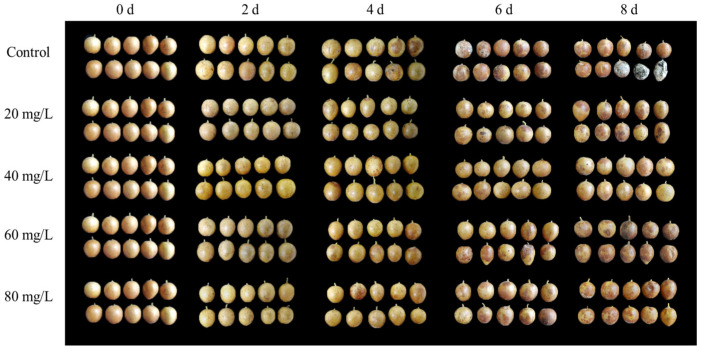
Effects of AEW on the fruit appearance quality in harvested wampees within storage.

In this study, the pericarp color of harvested wampee fruit was yellow on storage day 0 (Figure 1). Over the storage period, the pericarp color in the control samples gradually turned brown. Additionally, the control fruit’s surface developed white mycelium and exhibited pronounced symptoms of fruit disease and decay after day 4 (Figure 1). Compared with the control group, all AEW-treated groups showed the suppressed development of pericarp browning and fruit disease, with higher fruit appearance quality in wampees. Notably, wampees treated with AEW containing 40 mg/L ACC showed the best fruit appearance quality throughout storage. Therefore, AEW (especially with 40 mg/L ACC) enhanced harvested wampee fruit appearance quality during storage.

### 3.2. Changes in Fruit Storability

**Figure 2:**
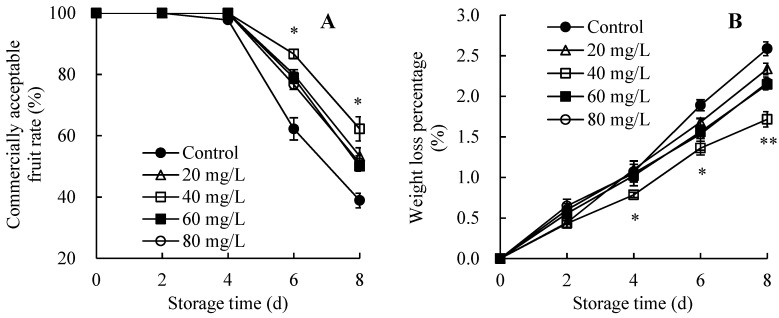
Effects of AEW on the commercially acceptable fruit rate (**A**) and weight loss percentage (**B**) in harvested wampees. Value in the figure shown as the mean ± standard error (*n* = 3), vertical bar shows the standard error. On same storage day, compared to control wampees, the conspicuous discrepancies in AEW-treated wampees are severally indicated via * (*p* < 0.05) or ** (*p* < 0.01). ●, control group; △, AEW (ACC = 20 mg/L)-treated group; □, AEW (ACC = 40 mg/L)-treated group; ■, AEW (ACC = 60 mg/L)-treated group; ○, AEW (ACC = 80 mg/L)-treated group.

Figure 2A showed that the rapid decline in the commercially acceptable fruit rate was observed in the non-AEW-treated group on days 2–8. In contrast, the AEW-treated group showed a higher commercially acceptable fruit rate within 2–8 days, with the optimal concentration being 40 mg/L ACC. Additionally, during days 6–8, the AEW-treated group containing 40 mg/L ACC maintained a notably higher commercially acceptable fruit rate than the control group.

A sharp increase in weight loss percentage was represented in all five groups within 0–8 days (Figure 2B). However, the AEW-treated group containing 40 mg/L ACC maintained a lower level than other treatments and displayed a markedly lower value than the control group during days 4–8.

Therefore, AEW (particularly with 40 mg/L ACC) could sustain a higher commercially acceptable fruit rate with a lower weight loss percentage in harvested wampees.

### 3.3. Changes in Chromaticity L^*^ and Hue Angle h Values

**Figure 3:**
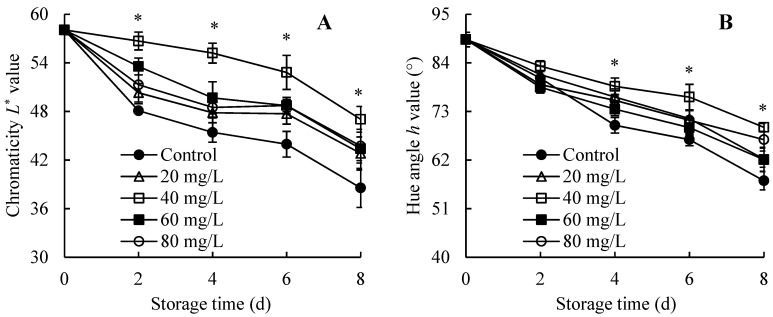
Effects of AEW on the levels of chromaticity L^*^ (**A**) and hue angle *h* (**B**) in harvested wampees. Value in the figure shown as the mean ± standard error (*n* = 3), vertical bar shows the standard error. On same storage day, compared to control wampees, the conspicuous discrepancies in AEW-treated wampees are severally indicated via * (*p* < 0.05). ●, control group; △, AEW (ACC = 20 mg/L)-treated group; □, AEW (ACC = 40 mg/L)-treated group; ■, AEW (ACC = 60 mg/L)-treated group; ○, AEW (ACC = 80 mg/L)-treated group.

A rapid decline in chromaticity L^*^ (Figure 3A) and hue angle *h* (Figure 3B) values in wampee pericarps were observed during days 0–8. Regarding the chromaticity L^*^ value, all AEW-treated groups maintained higher levels within 0–8 days than the control group, with the optimal concentration in the AEW group being 40 mg/L ACC. Furthermore, fruit treated with this concentration exhibited notably higher chromaticity L^*^ level than the control fruit within days 2–8. Regarding hue angle *h* value, fruit treated with AEW (ACC = 20 mg/L) and AEW (ACC = 40 mg/L)-treated fruit maintained higher levels within 0–8 d, but other AEW-treated groups maintained higher levels on days 4–8. Moreover, during days 4–8, samples treated with AEW containing 40 mg/L ACC showed conspicuously higher hue angle *h* values than the control samples.

Consequently, AEW (especially with 40 mg/L ACC) might maintain higher chromaticity L^*^ and hue angle *h* values in harvested wampees.

### 3.4. Changes in Pulp Nutrient Content

**Figure 4:**
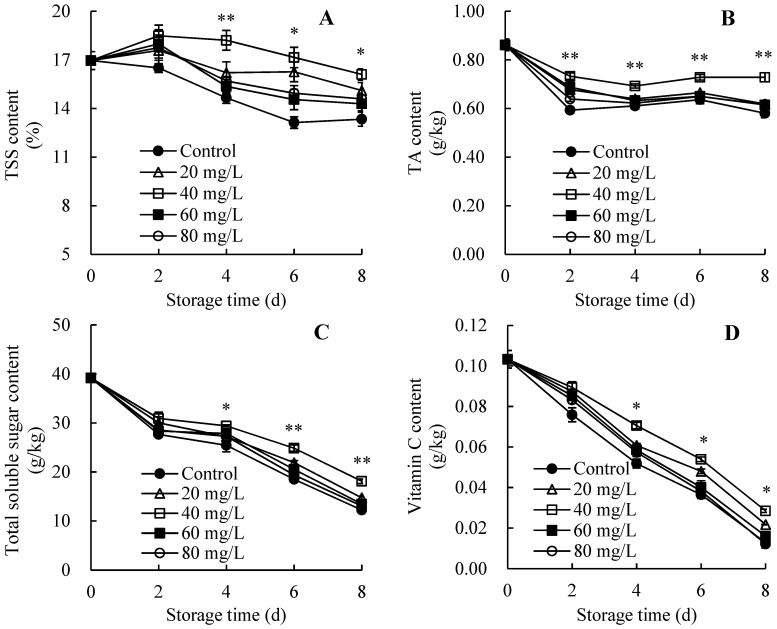
Effects of AEW on the pulp nutrient contents in harvested wampees. (**A**) TSS content; (**B**) TA content; (**C**) Total soluble sugar content; (**D**) Vitamin C content. Value in the figure shown as the mean ± standard error (*n* = 3), vertical bar shows the standard error. On same storage day, compared to control wampees, the conspicuous discrepancies in AEW-treated wampees are severally indicated via * (*p* < 0.05) or ** (*p* < 0.01). ●, control group; △, AEW (ACC = 20 mg/L)-treated group; □, AEW (ACC = 40 mg/L)-treated group; ■, AEW (ACC = 60 mg/L)-treated group; ○, AEW (ACC = 80 mg/L)-treated group.

Figure 4A showed that the overall trend of TSS content in five groups exhibited a downward trend on days 0–8. The AEW-treated groups, especially the 40 mg/L ACC group, demonstrated higher TSS level compared with the control group on days 0–8. Furthermore, on days 4–8, a substantially higher TSS level was manifested in the AEW-treated group containing 40 mg/L ACC compared with the control group.

The TA content in all groups exhibited a similar trend, displaying a dynamic downward trend on days 0–8 (Figure 4B). Higher TA content was consistently measured in AEW-treated groups throughout storage, especially in the group with 40 mg/L ACC. Further comparison revealed that wampees treated with AEW containing 40 mg/L ACC showed prominently higher TA content compared with control wampees on days 2–8.

Values of total soluble sugar (Figure 4C) and vitamin C (Figure 4D) sharply decreased in all treated wampees during storage. However, AEW-treated samples maintained higher levels of these two pulp nutrients, particularly in the AEW group with 40 mg/L ACC. Moreover, substantial differences in the values of total soluble sugar and vitamin C between the control fruit and fruit treated with AEW (40 mg/L ACC) were observed on days 4–6.

Hence, AEW treatment (particularly with 40 mg/L ACC) might result in higher pulp nutrient levels in harvested wampees.

### 3.5. Changes in the Fruit Disease Index and Pericarp Lignin Content

Given that 40 mg/L ACC in AEW was the most effective treatment for enhancing postharvest quality in harvested wampees, further investigation focused on the mechanism by which this AEW treatment slowed down fruit disease and improved storage quality by mediating disease resistance metabolism.

**Figure 5:**
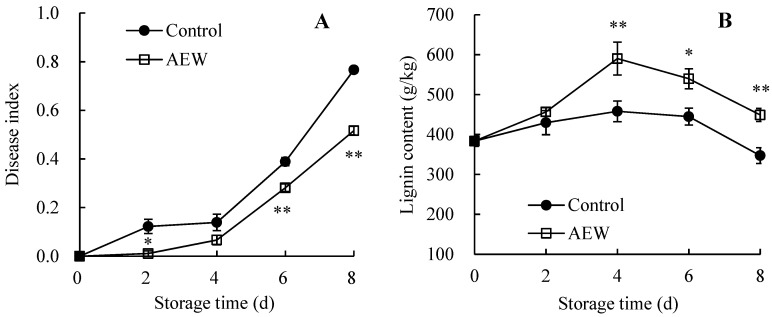
Effects of AEW on disease index (**A**) and pericarp lignin content (**B**) in harvested wampees. Value in the figure shown as the mean ± standard error (*n* = 3), vertical bar shows the standard error. On same storage day, compared to control wampees, the conspicuous discrepancies in AEW-treated wampees are severally indicated via * (*p* < 0.05) or ** (*p* < 0.01). ●, control group; □, AEW-treated group.

The disease index in the control fruit showed an upward trend during days 0–8 (Figure 5A). In comparison, the AEW-treated group exhibited a lower index of fruit disease (Figure 5A). Based on statistical analysis, AEW-treated samples displayed a markedly lower disease index on day 2 and during days 6–8.

Figure 5B showed that the pericarp lignin amount in the control fruit increased rapidly on days 0–4, reduced gradually on days 4–6, and then declined sharply until day 8. Conversely, AEW-treated samples showed a rapid increase in pericarp lignin level during days 0–4, followed by a swift decline on days 4–8. Additionally, compared with control fruit, a markedly higher lignin level was observed in AEW-treated fruit on days 4–8. For instance, the pericarp lignin content in AEW-treated wampees was 1.29 times that of the control wampees.

Consequently, AEW treatment delayed disease development and increased pericarp lignin levels in harvested wampees throughout storage.

### 3.6. Changes in Pericarp PAL, C4H, 4-CL, CAD, and PPO Activities

**Figure 6:**
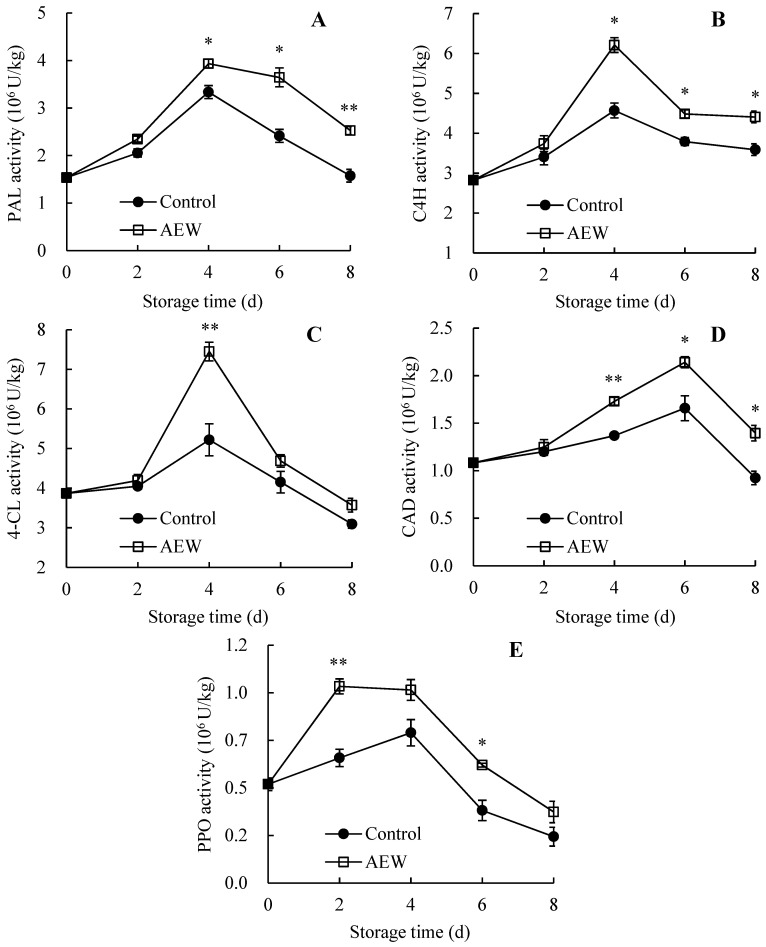
Effects of AEW on the activities of PAL (**A**), C4H (**B**), 4-CL (**C**), CAD (**D**) and PPO (**E**) in pericarp in harvested wampees. Value in the figure shown as the mean ± standard error (*n* = 3), vertical bar shows the standard error. On same storage day, compared to control wampees, the conspicuous discrepancies in AEW-treated wampees are severally indicated via * (*p* < 0.05) or ** (*p* < 0.01). ●, control group; □, AEW-treated group.

The activities of pericarp PAL (Figure 6A), C4H (Figure 6B), and 4-CL (Figure 6C) increased during days 0–4 but showed a declining trend during days 4–8 in both groups. In contrast, AEW-treated samples exhibited consistently higher activities of these enzymes on days 0–8. Regarding pericarp PAL and C4H activities, AEW-treated fruit maintained markedly higher values compared with control wampees on days 4–8. Regarding pericarp 4-CL, the AEW-treated group showed a conspicuously higher level on day 4. For instance, pericarp PAL, C4H, and 4-CL activities in AEW-treated wampees were 1.60, 1.23, and 1.16 times of the control fruit on day 8.

As shown in Figure 6D, the pericarp CAD activity of both groups increased rapidly during days 0–6 but declined quickly after day 6. However, the AEW-treated group sustained a higher level on days 0–8. Furthermore, during days 4–8, markedly higher pericarp CAD activity was observed in AEW-treated samples than in control samples. For example, the pericarp CAD activity in AEW-treated wampees was 1.51 times of the control fruit.

As depicted in Figure 6E, the pericarp PPO activity in the control group increased until day 4 and subsequently decreased from 0.76 × 10^6^ U/kg on day 4 to 0.23 × 10^6^ U/kg on day 8. In contrast, the pericarp PPO activity in AEW-treated wampee fruit increased sharply during days 0–2 before declining from 0.99 × 10^6^ U/kg on day 2 to 0.36 × 10^6^ U/kg on day 8. Furthermore, compared with control fruit, the AEW-treated fruit exhibited notably higher pericarp PPO activity on days 2 and 6.

Consequently, throughout storage, AEW enhanced the activities of pericarp PAL, C4H, 4-CL, CAD, and PPO in harvested wampee fruit.

### 3.7. Changes in Pericarp GLU and CHI Activities

**Figure 7:**
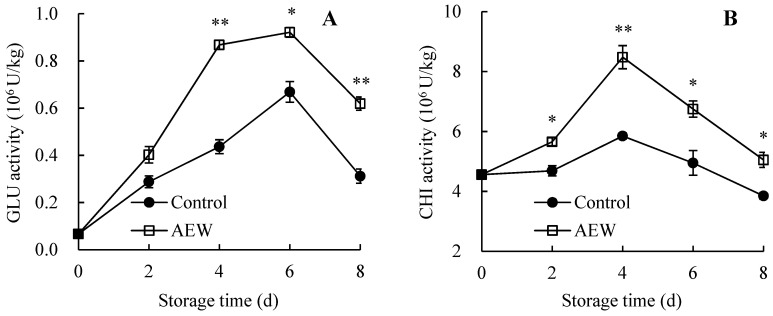
Effects of AEW on the activities of GLU (**A**) and CHI (**B**) in pericarp in harvested wampees. Value in the figure shown as the mean ± standard error (*n* = 3), vertical bar shows the standard error. On same storage day, compared to control wampees, the conspicuous discrepancies in AEW-treated wampees are severally indicated via * (*p* < 0.05) or ** (*p* < 0.01). ●, control group; □, AEW-treated group.

Pericarp GLU activity in the control group rose from 0.07 × 10^6^ U/kg (day 0) to 0.67 × 10^6^ U/kg (day 6) before declining to 0.31 × 10^6^ U/kg (day 8) (Figure 7A). However, pericarp GLU activity in AEW-treated samples rose from 0.07 × 10^6^ U/kg (day 0) to 0.92 × 10^6^ U/kg (day 6), followed by a decline to 0.62 × 10^6^ U/kg (day 8). Additionally, during days 4–8, AEW-treated samples exhibited remarkably higher GLU activity in the pericarp than the control samples.

Figure 7B showed that the pericarp CHI activity of the control group increased from 4.56 × 10^6^ U/kg on day 0 to 5.85 × 10^6^ U/kg on day 4 but declined to 3.85 × 10^6^ U/kg on day 8. However, the pericarp CHI activity of AEW-treated fruit rose from 4.56 × 10^6^ U/kg (day 0) to 8.48 × 10^6^ U/kg (day 4) before declining to 5.05 × 10^6^ U/kg (day 8). Additionally, during days 2–8, AEW-treated fruit exhibited markedly higher pericarp CHI activity than the control fruit.

Overall, AEW treatment might increase pericarp CHI and GLU activities of harvested wampees during storage.

## 4. Discussion

Harvested wampee fruit is susceptible to severe quality degradation after harvest, including disease development, pericarp browning, softening, and decay, all of which hinder the maintenance of fruit quality [1,4]. Previous studies have indicated that AEW serves as a safe, novel, and environmentally friendly technology for improving the quality of fresh produce [16,17,19]. Furthermore, the storage quality of fresh produce is affected by storage properties, pericarp appearance quality, and pulp nutrient levels [22,23]. Key indices of storability, such as commercially acceptable fruit rate and weight loss percentage, affect the quality of fresh produce [19,22,25]. Pericarp chromaticity parameters, such as L^*^ and hue angle *h*, contribute to evaluating the appearance quality of fresh fruit [19,23,25]. Nutrient levels in the pulp of fresh produce are associated with TA, total soluble sugar, TSS, and vitamin C contents [22,23].

In the present study, AEW application improved the quality of harvested wampees in comparison with the control wampees. These improvements were evident through enhanced storage properties, pericarp appearance quality, and pulp nutrient levels, as indicated by the elevated commercially acceptable fruit rate (Figure 2A), increased levels of chromaticity L^*^ and hue angle *h* (Figure 3), elevated total soluble sugar, TSS, vitamin C, and TA contents (Figure 4), and reduced weight loss level (Figure 2B) in wampees during storage. Upon closer comparison, AEW with 40 mg/L ACC resulted in an optimal enhancement of storage quality during storage. Therefore, this concentration was selected for further research on mitigating fruit disease and stabilizing storage quality in harvested wampees by mediating disease resistance metabolism. Similarly, Lin et al. [22] reported that chitosan-enhanced longan postharvest quality was attributed to chitosan’s impact on the rate of commercially acceptable fruit and levels of TSS, vitamin C, TA, and total soluble sugar. Additionally, melatonin application stabilized pericarp appearance quality, fruit storability, and pulp nutrient levels to enhance the quality of cold-stored guavas [23].

The lignin biosynthesis pathway constitutes a critical secondary metabolic pathway in the plant defense system [10]. Lignin, a phenolic polymer, plays a vital role in plant secondary cell walls, enhancing the mechanical strength of these walls and enhancing plant resistance to pathogen invasion [10,26,27]. Higher lignin content has been associated with increased disease defense, slowing disease development in plants [7,12,26]. Various treatments, including *ε*-poly-*l*-lysine [7], melatonin [10], ultraviolet-C irradiation [28], and burdock fructooligosaccharide [29], have been shown to promote lignin accumulation, which enhances disease resistance, slows disease occurrence, and stabilizes fresh product quality. Therefore, lignin content is crucial for postharvest disease occurrence, and higher lignin content may delay disease development while preserving the quality of fresh produce.

In the present study, AEW-treated wampee fruit displayed less severe disease symptoms (Figure 1) and a lower disease index (Figure 5A) compared with control wampees during storage. Additionally, AEW-treated wampees maintained a higher pericarp lignin amount compared with control wampees during days 0–8 (Figure 5B). Therefore, AEW delayed the fruit disease occurrence and stabilized the quality of harvested wampees by increasing pericarp lignin content, thereby enhancing disease resistance in harvested wampee fruit. These findings align with research demonstrating that caffeic-acid-treated apple fruit exhibited lower disease incidence due to the increased lignin content and enhanced disease resistance [30]. Additionally, ultraviolet-C irradiation could suppress disease occurrence in nectarine fruit by increasing lignin content [28]. Furthermore, Yu et al. [12] reported that chitooligosaccharide treatment might increase lignin content, induce disease resistance, and ultimately reduce disease development in pears.

Additionally, the DREs, including PAL, CAD, PPO, CHI, C4H, 4-CL, and GLU, play pivotal roles in plant disease resistance [5,7,30,31]. For example, PAL serves as the initial enzyme in lignin biosynthesis [15,29], influencing lignin level and regulating disease resistance [7]. C4H, 4-CL, and CAD are important DREs involved in lignin biosynthesis and metabolism [13,29,30]. Additionally, PPO converts phenolic compounds into quinones [15,27], which are highly toxic to pathogens, potentially leading to their elimination [9,32]. Moreover, GLU and CHI are key defense enzymes in plants [9,28,33], enhancing disease resistance by hydrolyzing *β*-1,3-glucan and chitin in pathogen cell walls [9,34].

Previous studies indicated that applications of chitosan [12,14], antimicrobial peptide CB-M [34], nitric oxide [6], and *β*-aminobutyric acid [35] could increase DRE activities, inducing boosted disease resistance and delaying disease occurrence in fresh products. Therefore, higher DRE activities are beneficial in reducing the disease symptoms and preserving the quality of fresh produce.

In the present study, AEW-treated wampee fruit displayed greater activities of PAL (Figure 6A), C4H (Figure 6B), 4-CL (Figure 6C), CAD (Figure 6D), PPO (Figure 6E), GLU (Figure 7A), and CHI (Figure 7B) in the pericarp throughout storage than the control fruit. Additionally, AEW-treated samples showed a higher pericarp lignin level (Figure 5B), less severe disease symptoms (Figure 1), and a lower disease index (Figure 5A) than the control samples during storage. These results suggested that AEW application promoted pericarp lignin accumulation by increasing the activities of PAL, C4H, 4-CL, and CAD, thereby enhancing disease resistance and subsequently delaying disease development in harvested wampee fruit. Additionally, AEW treatment might elevate PPO activity to induce the generation of quinones from phenolics and subsequently produce toxic effects for pathogens, leading to delayed disease incidence in harvested wampees. Furthermore, AEW treatment might disrupt pathogen cell walls by enhancing the actions of GLU and CHI, ultimately controlling fruit disease in harvested wampees. These findings indicated that AEW treatment on harvested wampee samples improved disease resistance and preserved storage quality by raising DRE activity levels. Similarly, ultraviolet-C irradiation increased the activities of GLU, PAL, and CHI, enhancing disease resistance and reducing disease incidence in mangosteen fruit [36]. Methionine treatment also delayed disease development in jujube fruit due to the increased activities of DREs, such as PAL and 4-CL [10].

**Figure 8:**
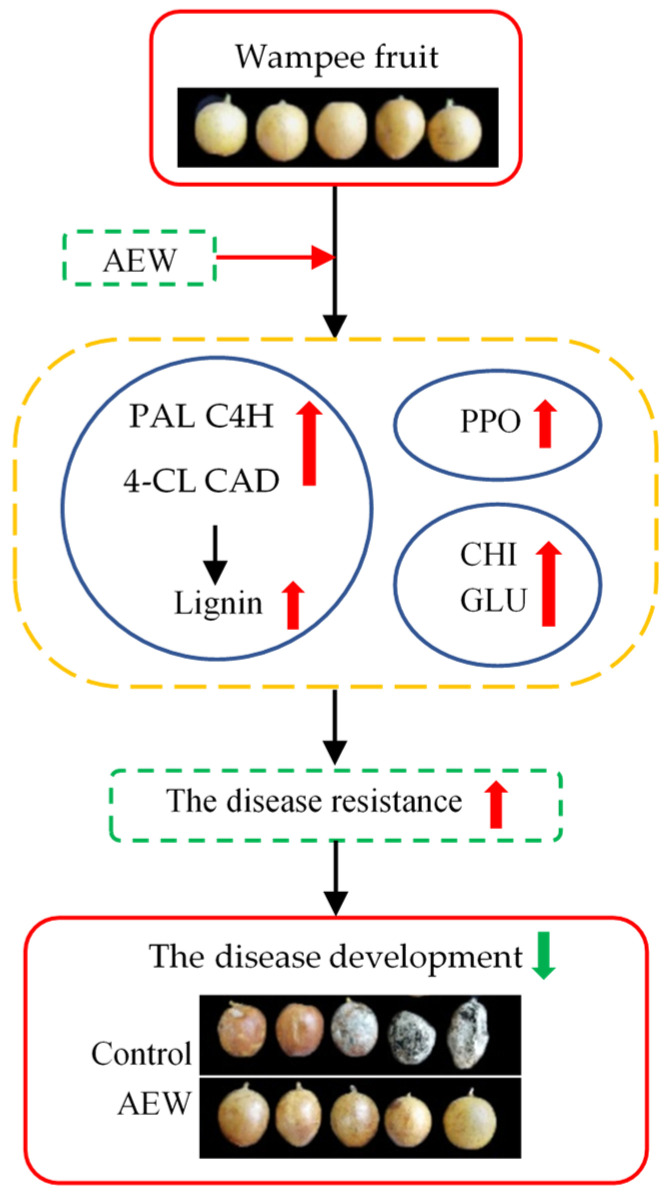
The possible mechanism of AEW suppressing the disease development and improving the storage quality in harvested wampee fruit through modulating the disease resistance metabolism.

Therefore, the probable mechanism of disease resistance metabolism underlying AEW’s ability to postpone the development of fruit disease and maintain the storage quality in harvested wampees is illustrated in Figure 8.

## 5. Conclusions

In summary, AEW treatment demonstrated a potential suppressive effect on disease development while maintained higher storage quality in harvested wampee fruit during storage. AEW also delayed the incidence of fruit disease in harvested wampees by increasing the activities of DREs (PAL, PPO, CHI, 4-CL, GLU, C4H, and CAD) and raising lignin accumulation, thereby enhancing disease resistance, delaying disease development, and maintaining storage quality. Future works might research the molecular mechanisms of AEW with 40 mg/L ACC in mitigating disease incidence and improving quality in harvested wampees, using proteomics, transcriptomics, and metabolomics.

## Data Availability

The original contributions presented in the study are included in the article, further inquiries can be directed to the corresponding authors.
